# Synergistic Effects of Six Chronic Disease Pairs on Decreased Physical Activity: The SMILE Cohort Study

**DOI:** 10.1155/2016/9427231

**Published:** 2016-05-05

**Authors:** Sarah Dörenkamp, Ilse Mesters, Rein Vos, Jan Schepers, Marjan van den Akker, Joep Teijink, Rob de Bie

**Affiliations:** ^1^Department of Epidemiology and CAPHRI School for Public Health and Primary Care, Functioning and Rehabilitation Programme, Maastricht University, 6229 ER Maastricht, Netherlands; ^2^Department of Methodology and Statistics and CAPHRI School for Public Health and Primary Care, Maastricht University, 6229 ER Maastricht, Netherlands; ^3^Department of Medical Informatics, Erasmus University Rotterdam, 3015 CE Rotterdam, Netherlands; ^4^Department of Family Medicine and CAPHRI School for Public Health and Primary Care, Maastricht University, 6229 ER Maastricht, Netherlands; ^5^Department of General Practice, Catholic University Leuven, 3000 Leuven, Belgium; ^6^Department of Vascular Surgery, Catharina Hospital, 5623 EJ Eindhoven, Netherlands

## Abstract

Little is known about whether and how two chronic diseases interact with each other in modifying the risk of physical inactivity. The aim of the present study is to identify chronic disease pairs that are associated with compliance or noncompliance with the Dutch PA guideline recommendation and to study whether specific chronic disease pairs indicate an extra effect on top of the effects of the diseases individually. Cross-sectional data from 3,386 participants of cohort study SMILE were used and logistic regression analysis was performed to study the joint effect of the two diseases of each chronic disease pair for compliance with the Dutch PA guideline. For six chronic disease pairs, patients suffering from both diseases belonging to these disease pairs in question show a higher probability of noncompliance to the Dutch PA guideline, compared to what one would expect based on the effects of each of the two diseases alone. These six chronic disease pairs were chronic respiratory disease and severe back problems; migraine and inflammatory joint disease; chronic respiratory disease and severe kidney disease; chronic respiratory disease and inflammatory joint disease; inflammatory joint disease and rheumatoid arthritis; and rheumatoid arthritis and osteoarthritis of the knees, hips, and hands.

## 1. Introduction

Inspired by Health 2020 [1], the first World Health Organization (WHO) strategy to tackle physical inactivity in the European Region from 2016 to 2025 was released last September. The aim of this strategy is to inspire governments and stakeholders to promote physical activity (PA) levels among all citizens in the European Region. The rationale for this effort was that health care systems are at risk of being inundated by large numbers of people suffering the effects of physical inactivity and sedentary behaviour, such as coronary heart disease, hypertension, colon cancer, and diabetes mellitus [2]. Suffering from a disease is a risk factor in the downward spiral of PA, especially among those suffering from more than one chronic disease [3, 4].

To date, most studies that investigated the association between chronic disease and PA are limited to one chronic disease in particular, thus avoiding the actual complexity [5–8]. From previous literature we know that patients with chronic diseases and/or multimorbidity are at risk for physical inactivity [1–4]. For clinicians and physical therapists it is particularly important to identify patients with specific combinations of chronic diseases that are at risk for extra physical inactivity, so that more conscious approaches to initiate or increase physical activity in these patients can be applied. Although most chronic diseases are associated with physical inactivity, little is known about whether two chronic diseases might interact with each other in modifying the risk of physical inactivity. Since the effect of two chronic diseases on physical inactivity might not always be simply additive it is important to highlight those chronic disease pairs for whom the effect of both diseases on physical inactivity is greater than the sum of the individual effects alone. Our main research interest was to investigate whether certain disease pairs/combinations indicate an “extra” additional effect, on top of the effects of the diseases separately/individually. We hypothesized that exposure to a specific combination of chronic diseases had additional/extra (interaction) effects on the risk of inadequate physical activity levels above what would be expected from either exposure alone.

The present study has two aims, based on the observation that chronic disease pairs provide information about how combinations of diseases behave and how they may be associated with PA. The first aim is to identify chronic disease pairs that are associated with compliance or noncompliance with the Dutch PA guideline recommendation and the second is to investigate whether specific chronic disease pairs indicate an additional/extra (interaction) effect on top of the diseases separately/individually. A portion of the data from the large prospective cohort Study of Medical Information and Lifestyles in Eindhoven (SMILE), Netherlands, was used for the present study [9].

## 2. Method

### 2.1. Study Design and Setting

The present study used a portion of the data from the prospective dynamic cohort SMILE, Netherlands [9]. The SMILE study focuses on different aspects of disease, health, and lifestyle and is a joint project between Maastricht University and 23 general practitioners from nine primary health care centers of the Eindhoven Corporation of Primary Health Care Centers (Stichting Gezondheidscentra Eindhoven (SGE)) in Eindhoven. The SMILE cohort study was conducted between 2002 and 2010. One goal of SMILE is to study diseases and their consequences. In the SMILE cohort study two data collection strategies were combined. With the help of electronic medical records (EMRs), data on morbidity, mortality, medication, and care facility utilisation were registered. Information on lifestyle and chronic diseases was gathered on the basis of patient questionnaires which were self-administered and completed on paper. Detailed information on the study protocol can be found in a publication by van den Akker et al. [9]. The SMILE protocol was approved by the medical ethics committee of the Academic Hospital Maastricht (MEC 07-04-030) and all participants signed written informed consent forms [9]. This paper was written according to STrengthening the Reporting of OBservational Studies in Epidemiology (STROBE) checklist for cohort studies in order to enhance transparency and reproducibility.

### 2.2. Participants

Data of adult patients aged 55 from 2003 were used, as the numbers of patients who returned their information on chronic diseases and PA behaviour (*n* = 3,386) were largest for that cohort year.

### 2.3. Data Sources/Measurement

All participants completed two questionnaires. Information on chronic diseases was measured using a self-reported chronic disease questionnaire [10] which was distributed and returned in May 2003. Physical activity behaviour was measured using the Short Questionnaire to Assess Health-Enhancing Physical Activity (SQUASH) [11]. Data from the SQUASH was gathered in November 2003.

#### 2.3.1. Self-Reported Chronic Disease Questionnaire

Information about the presence/absence of chronic disease was obtained from the self-reported chronic disease questionnaire, for which the medical screening questionnaire of the Dutch Association of General Practitioners (Landelijke Huisartsen Vereniging (LHV)) served as a template [8]. Participants were asked to report whether they had any of the following fifteen chronic diseases at that time: (1) chronic respiratory disease; (2) cardiovascular disease; (3) severe bowel disease; (4) liver disease; (5) severe kidney disease; (6) diabetes mellitus; (7) cancer; (8) epilepsy; (9) migraine; (10) neurological disorders and stroke; (11) inflammatory joint disease; (12) rheumatoid arthritis; (13) osteoarthritis of the knees, hips, and hands; (14) severe back problems; and (15) persistent injuries due to accidents. Furthermore, in response to an open question, patients could report other diseases they had which were not listed in the questionnaire. The chronic diseases mentioned in response to this open question (*N* = 1,077) were integrated into the questionnaire data. Two researchers (SD and IM) and one medical specialist (JT) independently assigned each disease to the existing categories of the chronic disease questionnaire.

#### 2.3.2. Short Questionnaire to Assess Health-Enhancing Physical Activity (SQUASH)

The SQUASH was used to measure PA [11]. This questionnaire asked about three main items: (1) how many days per week one was active, (2) average time of activity per day, and (3) PA intensity. Therefore it was possible to assess compliance with the Dutch PA guideline with this questionnaire. Participants were asked to report their level of PA during an average week over the past few months. To simplify reporting, the questionnaire was subdivided into (a) commuting activities, (b) leisure time activities, (c) household activities, and (d) activities at work and at school. Using the Ainsworth compendium for physical activities, a MET value was assigned to each activity [12, 13]. Activities between 1.6 and 2.9 METs were classified as light-intense, activities between 3.0 and 5.9 METs as moderate-intense, and activities ≥ 6 METs as vigorous-intense [13]. To determine compliance to the Dutch PA guideline, the frequency, duration, and intensity of each activity were multiplied by each other.

### 2.4. Variables

Chronic disease pairs were used as an independent variable. Disease pairs were constructed based on the fifteen chronic diseases listed in the self-reported chronic disease questionnaire. All possible combinations of two chronic diseases were established. The primary outcome was compliance with the Dutch PA guideline (0 = compliance with the guideline and 1 = lack of compliance with the guideline). This guideline states that all adults should participate in a total of at least 30 minutes of moderate-intense PA at least five days a week, but preferably daily [14].

### 2.5. Bias

Of the 15 chronic diseases listed in the self-reported chronic diseases questionnaire, missing values amounting to some 17.5% to 24.2% were detected. The format of the questionnaire was dichotomous and prestructured (yes/no). The hypothesis is that a proportion of respondents followed these instructions to the letter and only indicated the diseases they had without explicitly indicating which ones they did not suffer from (i.e., by ticking “no”). Therefore, all missing values were recorded as “disease being absent.”

### 2.6. Statistical Analysis

**Figure 1:**
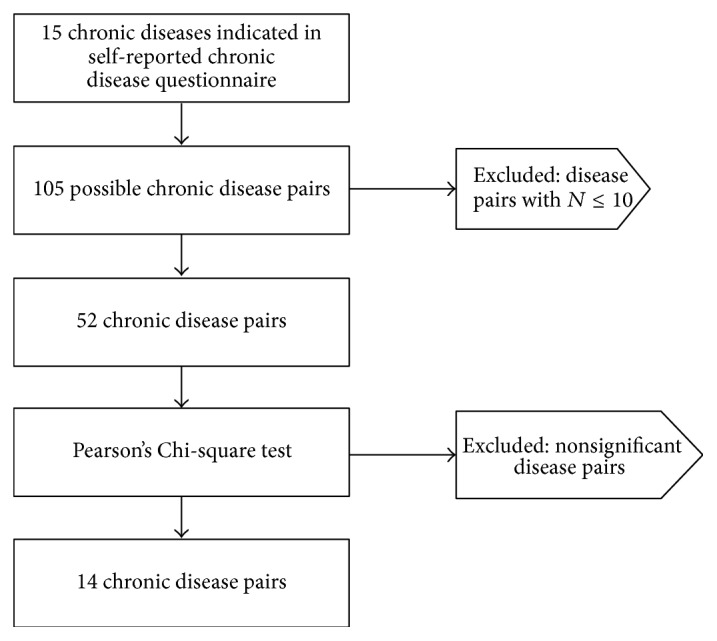
Flowchart of the selection of chronic disease pairs.

First, from the fifteen chronic diseases listed in the self-reported chronic disease questionnaire, a number of 105 (*N∗*[(*N* − 1)/2]) disease pairs (disease A + disease B) were calculated (Table 5). The occurrence of diseases pairs was calculated and pairs that affected fewer than 10 participants were excluded from the analysis in order to maximise the chance of showing an association with compliance to the Dutch PA guideline. Pearson's Chi-square test was used to test whether patients having the combination of two diseases show higher probabilities of noncompliance compared to the other patients. The alpha was set at <0.10 to give room for the detection of all chronic disease pairs associated with PA that might be relevant for clinical practice. For all cross tables with a value *N* ≤ 5 in at least one of the cells, Fisher's exact test was used as alternative for the Chi-square [15]. Significant chronic disease pairs were listed and were the subject of further analysis (*N* = 14; Figure 1).

The second aim of the present study was to study whether the association between chronic disease pairs and PA guideline compliance surpasses the expected result of the combined effects of both diseases individually. Logistic regression analyses were performed to study the joint effect of the two diseases of each chronic disease pair for compliance with the Dutch PA guideline. Compliance with the Dutch PA guideline (1 = lack of compliance with the guideline; 0 = compliance with the guideline) was used as a dependent variable. Independent variables included the two indicator variables that represent occurrence of the two diseases of the pair as well as the product of these two indicator variables in order to capture a potential interaction effect. Main effects of each disease separately/individually are not presented but can be read from the model: the simple main effect of A (in terms of an odds ratio) for B = 1 is equal to exp(coefficient  of  A)*∗*exp(coefficient  of  the  interaction  term); the simple main effect of B (in terms of an odds ratio) for A = 1 is equal to exp(coefficient  of  *B*)*∗*exp(coefficient  of  the  interaction  term). Chronic disease pairs significant at *p* < 0.10 were studied.

## 3. Results

### 3.1. Participants

In total 3,386 participants (52.9% female) completed and returned the self-reported chronic disease questionnaire and the SQUASH. The average age of respondents was 67.5 years of age (range: 55–95 years). Forty-seven percent of the participants did not suffer from any chronic disease, while 28%, 14%, 7%, 3%, and 1% reported having one, two, three, four, and five chronic diseases, respectively (Table 1).

### 3.2. Selection of Chronic Disease Pairs

In total 105 possible chronic disease pairs were assembled from the 15 chronic diseases indicated in the self-reported chronic disease questionnaire (Tables 2 and 5). Fifty-three pairs were excluded because ten or fewer participants suffered from them. The most common chronic disease pair was “osteoarthritis of knees, hips, and hands” and “severe back problems” (*N* = 259). Other chronic disease pairs affecting more than one hundred participants were “inflammatory joint disease” and “osteoarthritis of knees, hips, and hands” (*N* = 194); “chronic respiratory disease” and “osteoarthritis of knees, hips, and hands” (*N* = 114); and “inflammatory joint disease” and “severe back problems” (*N* = 109).

For each of the 52 chronic disease pairs that were selected for further study, it was tested whether patients having the combination of the two diseases show higher probabilities of noncompliance compared to the other patients. This was the case for fourteen chronic disease pairs (Table 3). Chronic respiratory disease seemed to cooccur in five of the pairs. Cardiovascular disease, inflammatory joint disease, rheumatoid arthritis, and diabetes mellitus were part of four disease pairs. Osteoarthritis of knees, hips, and hands emerged in three disease pairs. The four remaining diseases forming part of disease pairs were severe kidney disease, migraine, back problems, and persistent injury due to an accident (Table 3).

### 3.3. Association between Chronic Disease Pairs and Lack of Compliance with PA Guideline

Table 3 demonstrated that these 14 chronic disease pairs are particularly interesting, because patients suffering from both diseases of these pairs show a higher probability of noncompliance to the PA guideline compared to other patients. Therefore, we included these 14 chronic disease pairs in the logistic regression analysis. The results showed that the interaction term of six chronic disease pairs was statistically significant, which means that the effect of the two diseases combined is not additive. Furthermore, in all six cases the odds ratio of the interaction term is larger than one, which means that patients suffering from both diseases of the pair in question have an increased risk for noncompliance with the Dutch PA guideline recommendation compared to what one would expect based on the effects of each of the two diseases alone (Table 4).

Patients who have the chronic disease pair chronic respiratory disease and severe back problems have an even higher risk for noncompliance to the Dutch PA guideline recommendation than expected on the effect of the diseases individually. The same was found for the chronic disease pair migraine and inflammatory joint disease. Patients that have both migraine and inflammatory joint disease have an extra risk to show inadequate PA levels. Four other chronic disease pairs were found to be particularly associated with noncompliance with the PA guideline at a borderline significance level. Because they might still be relevant for clinical practice we decided to present them, as well. The first chronic disease pair was chronic respiratory disease and severe kidney disease. These patients have an extra risk of a lack of PA guideline compliance. The same applies to patients who suffered from chronic respiratory disease and inflammatory joint disease simultaneously. Patients with the chronic disease pair rheumatoid arthritis and inflammatory joint disease, as well as rheumatoid arthritis and osteoarthritis of the knees, hips, and hands also have an additional risk to not show adequate PA behaviour (Table 4).

**Figure 2:**
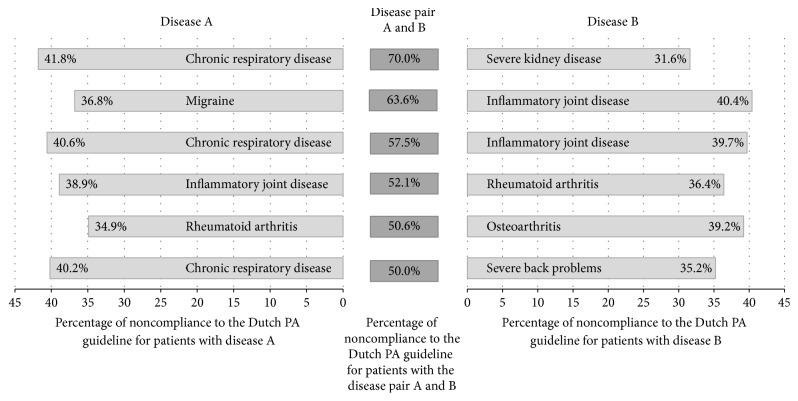
Percentage of participants with disease A, disease B, and/or the disease pair A and B who did not comply with the Dutch physical activity guideline.

Figure 2 presents the percentage of participants with disease A, disease B, and the chronic disease pair A and B for whom the probability of noncompliance with the Dutch PA guideline is larger than compared to what one would expect from the effects of each of the two diseases alone. Each disease pair consists of disease A and disease B. The left and the right side of Figure 1 present the percentage of noncompliance with the Dutch PA guideline for patients that suffered from disease A only, but not from disease B (left side), and for patients that suffered from disease B, but not from disease A (right side). For example, of the patients who had diabetes mellitus, but not rheumatoid arthritis, 34.9% did not comply with the Dutch PA guideline (disease A, left side). Of the patients who suffered from rheumatoid arthritis, but not diabetes mellitus, 39.2% did not comply with the Dutch PA guideline recommendation (disease B, right side). Of all patients who suffered from the chronic disease pair diabetes mellitus and rheumatoid arthritis (disease pair A + B, middle), 69.2% did not comply with the Dutch PA guideline. The strongest association of guideline noncompliance was found in patients with the disease pair diabetes mellitus and rheumatoid arthritis (percentage of noncompliance: 69.2%; odds ratio: 3.652).

## 4. Discussion

Six chronic disease pairs were shown to be particularly interesting, because patients suffering from both diseases belonging to these disease pairs in question show a higher probability of noncompliance to the Dutch PA guideline, compared to what one would expect based on the effects of each of the two diseases alone. These six chronic disease pairs were chronic respiratory disease and severe back problems; migraine and inflammatory joint disease; chronic respiratory disease and severe kidney disease; chronic respiratory disease and inflammatory joint disease; inflammatory joint disease and rheumatoid arthritis; and rheumatoid arthritis and osteoarthritis of the knees, hips, and hands.

Some limitations are worth mentioning. First, the presence or absence of chronic diseases was measured via self-reported questionnaires. In the SMILE cohort study, information on chronic diseases was also registered in EMRs; however not all participants gave written consent to allow comparing self-reported information with data registered in the EMRs by general practitioners. Nevertheless, previous research compared self-reported SMILE cohort data with EMR information and revealed an agreement of over 80% for most of the chronic diseases. This supports the use of self-reported data to answer our research question [16]. Secondly, to preserve as much information as possible, missing values in the self-reported chronic disease questionnaire were interpreted as “absence of disease.” This may have led to an underestimation of the total disease burden of the population. Thirdly, the self-reported chronic disease questionnaire provided information on fifteen chronic diseases without severity assessment, which could be interpreted as a limitation. Lastly, self-reported PA measurement could have been influenced by social desirability and seasonal factors. Previous research conducted by Sallis and Saelens [17] already showed that people tend to overestimate their PA. To limit this type of bias, participants were explicitly informed that neither researchers nor their caregivers would receive any information from the self-reported questionnaires.

An exceeded inverse association between the chronic disease pair chronic respiratory disease and severe back problems and Dutch PA guideline compliance was revealed. This chronic disease pair might extra interfere with PA due to the interrelated anatomical and physiological cohesive structure of the thoracic cage, which is formed by the spine, rib cage, and associated muscles, which are affected in myriad ways in patients with chronic respiratory disease and severe back problems [18]. One of the main symptoms of chronic respiratory disease, which was defined in the self-reported disease questionnaire as chronic bronchitis, emphysema, and asthma, is a hacking cough which may induce and aggravate severe musculoskeletal back pain. Furthermore, biochemical and neurological interactions between chronic respiratory disease and severe back problems might underpin the exceeded inverse association between this chronic disease pair and inadequate PA levels. A decrease in CO_2_ together with an increase in pH inhibits the transfer from haemoglobin of oxygen to tissue cells, which affects normal muscular function, motor control, and pain perception [18]. Taken together, the strength of the interdependency between chronic respiratory disease and severe back problems might worsen the pain and decrease functional ability of patients and thereby extra interfere with adequate PA behaviour.

Patients suffering from the chronic disease pair migraine and inflammatory joint disease also showed extraordinary inadequate PA levels. Previous research has shown that the association between migraine and PA is two-sided. On the one hand, PA has been shown to have a beneficial effect on the frequency, intensity, and duration of migraine attacks and patient well-being [19–21]. On the other hand, PA has been shown to be a common trigger for headaches and migraine attacks [22, 23]. Strenuous activities often cause dehydration, higher body temperature, low blood sugar, and therefore trigger migraine due to low blood oxygen levels [22]. Patients with inflammatory joint disease experience persistent joint pain and swelling of the joints due to the inflammatory process, which can also occur during PA [24]. Being more vigorous, strengthening exercises are often recommended for patients with inflammatory joint disease because they are designed to increase muscle strength. As the muscle becomes stronger, it provides greater joint support and thereby reduces stress and loading on the painful joint. Moreover, strong muscles contribute to better functioning. Therefore, patients that have both migraine and inflammatory joint disease may display particularly inadequate PA levels, because they try to avoid exercises that could trigger a migraine. When stiffness, painful joints, and headaches are already bogging patients down, the prospect of PA may seem overwhelming and painful, causing them to limit their activity.

At the 10% significance level it was found that the association between the chronic disease pair chronic respiratory disease and severe kidney disease with PA was exceedingly reversed. Severe kidney disease generally refers to the progressive and irreversible loss of kidney function, in which in the most severe stage renal replacement therapy in the form of either dialysis or kidney transplantation becomes necessary in order to keep the patient alive and maintain quality of life [25]. Physical activity levels in patients with severe kidney disease are usually low [26, 27]. Additionally, these patients report lower physical functioning and performance than those with normal kidney function [26]. Literature has shown that the burden of other chronic diseases in patients with more severe kidney disease is significantly higher [28]. Patients with the disease combination chronic respiratory disease and severe kidney disease may perceive themselves unable to participate in PA because of the disruptive and burdensome effect of both diseases on their daily life and well-being. Moreover, shortness of breath caused by chronic respiratory disease may worsen due to the additional influence of severe kidney disease. The extra fluid in the body can accumulate in the lungs. In addition, anaemia may lead to oxygen starvation and shortness of breath. The combination of both diseases may also aggravate fatigue. Fatigue, defined as the perception of mental or physical exhaustion, is a common symptom in chronic respiratory diseases [29]. As the kidney's fail, they produce less erythropoietin, a hormone that initiates the production of oxygen-carrying blood cells. Consequently, chronic respiratory disease in combination with fewer red blood cells carrying oxygen due to kidney failure leads to anaemia, which causes the muscles and brain to become exhausted very easily [28]. Besides, the physiological explanation for low PA levels in patients who suffer from respiratory disease and severe kidney disease must acknowledge the low statistical power of the analysis of this chronic disease pair (*N* = 10).

Moreover, patients with the chronic disease pair chronic respiratory disease and inflammatory joint disease combined showed extra inadequate PA levels. Previous research showed that joint inflammation might be interrelated with chronic respiratory disease [29]. Long-term inflammation may cause scarring of the lungs, which in turn leads to shortness of breath, chronic dry cough, and fatigue. Additionally, inflammation can also cause pleural inflammation, resulting in shortness of breath and painful respiration. The connectedness of both chronic diseases might increase symptom severity, especially pain perception and breathing problems which may in turn explain why patients with chronic respiratory disease and inflammatory joint disease avoid PA and are therefore eminently noncompliant with the Dutch PA guideline recommendation.

Last, two chronic disease pairs that show an ancillary inverse association with PA are rheumatoid arthritis and inflammatory joint disease and rheumatoid arthritis and osteoarthritis of the knees, hips, and hands. A phenomenological study conducted by Petursdottir et al. [30] identified facilitators and barriers to exercising among people with osteoarthritis. Patients suffering from osteoarthritis of the knees, hips, and hands often have difficulties with PA because of symptoms such as pain and stiffness. Furthermore, uncertainty about the amount and type of exercise they require, as well as when to expect benefits, seems to contribute to inadequate PA levels [30]. Prior negative experiences with overtraining resulting in increased pain levels after PA have been found in 15% of all patients with osteoarthritis and might contribute to PA guideline noncompliance [30]. Furthermore, it is well known that the pain levels of all individual diseases vary from day to day. The combination of two diseases might cause constant pain, which the patient may perceive as too intense to engage in PA. Moreover, perceived frailty and perceived poor health might play a role in patients with one of these two chronic disease pairs [31].

## 5. Conclusions

Six chronic disease pairs were identified in which having the combination of the two diseases leads to even more noncompliance than expected based on the sum of the diseases individually: chronic respiratory disease and severe back problems; migraine and inflammatory joint disease; chronic respiratory disease and severe kidney disease; chronic respiratory disease and inflammatory joint disease; inflammatory joint disease and rheumatoid arthritis; and rheumatoid arthritis and osteoarthritis of the knees, hips, and hands. The results of the present study may alert health care professionals of particularly low physical activity levels in patients with one of the six chronic disease pairs. Further research is needed to back up our findings to test whether these results remain stable in different patient populations.

## Figures and Tables

**Table 1:** Participant characteristics.

Characteristic^a^	Participants (*N* = 3,386)
Age (years)	67.5 ± 8.3
Sex (*N*, % female)	1791 (52.9)
Height (cm)	170.0 ± 8.8
Body weight (kg)	75.1 ± 13.8
Osteoarthritis of knees, hips, or hands	780 (23.0)
Severe back problems	517 (15.3)
Chronic respiratory disease	321 (9.5)
Inflammatory joint disease	302 (8.9)
Cardiovascular disease	299 (8.8)
Diabetes mellitus	230 (6.8)
Migraine	158 (4.7)
Rheumatoid arthritis	150 (4.4)
Persistent injury due to an accident	132 (3.9)
Severe bowel disease	112 (3.3)
Cancer	77 (2.3)
Neurological disorders and stroke	70 (2.1)
Severe kidney disease	48 (1.4)
Epilepsy	20 (0.6)
Liver disease	16 (0.5)

^a^Continuous variables are presented as mean ± standard deviation and dichotomous variables as *N* (%).

**Table 2:** Inclusion/exclusion of chronic disease pairs based on the prevalence of each chronic disease pair in 3386 participants.

*N* ^†^ = excluded *N* < 10 *N* ^‡^ = 52 included disease pairs	Chronic respiratory disease	Cardiovascular disease	Severe bowel disease	Liver disease	Severe kidney disease	Diabetes mellitus	Cancer	Epilepsy	Migraine	Neurological disorders and stroke	Inflammatory joint disease	Rheumatoid arthritis	Osteoarthritis of knees, hips, and hands	Severe back problems
Cardiovascular disease	47^‡^													
Severe bowel disease	10^‡^	6^†^												
Liver disease	3^†^	3^†^	0^†^											
Severe kidney disease	10^‡^	6^†^	1^†^	1^†^										
Diabetes mellitus	40^‡^	37^‡^	5^†^	4^†^	6^†^									
Cancer	12^‡^	15^‡^	4^†^	2^†^	6^†^	11^†^								
Epilepsy	5^†^	3^†^	0^†^	0^†^	0^†^	4^†^	3^†^							
Migraine	15^‡^	14^‡^	12^‡^	2^†^	2^†^	4^†^	9^†^	3^†^						
Neurological disorders and stroke	10^‡^	14^‡^	3^†^	0^†^	0^†^	7^†^	2^†^	2^†^	5^†^					
Inflammatory joint disease	40^‡^	40^‡^	15^‡^	8^†^	8^†^	33^‡^	11^‡^	3^†^	22^‡^	12^‡^				
Rheumatoid arthritis	24^‡^	19^‡^	6^†^	3^†^	3^†^	13^‡^	5^†^	1^†^	11^‡^	8^†^	73^‡^			
Osteoarthritis of knees, hips, and hands	114^‡^	86^‡^	30^‡^	20^‡^	20^‡^	73^‡^	19^‡^	9^†^	61^‡^	20^‡^	194^‡^	87^‡^		
Severe back problems	82^‡^	64^‡^	25^‡^	6^†^	6^†^	35^‡^	12^‡^	8^†^	47^‡^	14^‡^	109^‡^	54^‡^	259^‡^	
Persistent injury due to an accident	18^‡^	18^‡^	3^†^	2^†^	2^†^	12^‡^	3^†^	1^†^	14^‡^	2^†^	17^‡^	7^†^	53^‡^	42^‡^

**Table 3:** Chronic disease pairs inversely associated with PA guideline compliance.

Chronic disease pair	*N*	Chi-square	*p* value
Cardiovascular disease and osteoarthritis of knees, hips, and hands	86	7.602	0.006
Diabetes mellitus and osteoarthritis of knees, hips, and hands	73	6.371	0.012
Chronic respiratory disease and inflammatory joint disease	40	5.714	0.018
Migraine and inflammatory joint disease	22	5.568	0.018
Inflammatory joint disease and rheumatoid arthritis	73	5.206	0.024
Diabetes mellitus and rheumatoid arthritis	13	4.953	0.026
Rheumatoid arthritis and osteoarthritis of knees, hips, and hands	87	4.882	0.028
Cardiovascular disease and inflammatory joint disease	40	4.262	0.040
Chronic respiratory disease and severe back problems	82	4.144	0.042
Chronic respiratory disease and severe kidney disease	10	4.004	0.098
Chronic respiratory disease and cardiovascular disease	47	3.938	0.048
Diabetes mellitus and persistent injury due to an accident	12	3.824	0.100
Chronic respiratory disease and rheumatoid arthritis	24	3.729	0.054
Cardiovascular disease and diabetes mellitus	37	3.482	0.062

**Table 4:** Association between chronic disease pairs and PA guideline compliance.

Disease		*N*	Odds ratio	95% confidence interval		*p* value
				Lower	Upper	
A	Chronic respiratory disease	311	1.129	0.891	1.431	0.316
B	Severe kidney disease	38	0.725	0.365	1.443	0.360
A and B	Chronic respiratory disease and severe kidney disease	10	4.478	0.966	20.765	0.055^+^

A	Migraine	136	0.910	0.637	1.300	0.605
B	Inflammatory joint disease	280	1.059	0.825	1.360	0.651
A and B	Migraine and inflammatory joint disease	22	2.841	1.078	7.486	0.035^*∗*^

A	Chronic respiratory disease	281	1.081	0.842	1.388	0.542
B	Inflammatory joint disease	262	1.042	0.805	1.350	1.042
A and B	Chronic respiratory disease and inflammatory joint disease	40	1.902	0.927	3.902	0.080^+^

A	Inflammatory joint disease	229	0.997	0.757	1.313	0.981
B	Rheumatoid arthritis	77	0.896	0.560	1.434	0.647
A and B	Inflammatory joint disease and rheumatoid arthritis	73	1.906	0.938	3.873	0.074^+^

A	Rheumatoid arthritis	63	0.845	0.500	1.426	0.527
B	Osteoarthritis of knees, hips, and hands	693	1.017	0.856	1.208	0.849
A and B	Rheumatoid arthritis and osteoarthritis of knees, hips, and hands	87	1.875	0.942	3.735	0.074^+^

A	Chronic respiratory disease	239	1.033	0.788	1.353	0.814
B	Severe back problems	435	0.835	0.675	1.032	0.095
A and B	Chronic respiratory disease and severe back problems	82	1.784	1.033	3.083	0.038^*∗*^

A	Cardiovascular disease	213	1.247	0.940	1.656	0.126
B	Osteoarthritis of knees, hips, and hands	694	1.025	0.862	1.219	0.780
A and B	Cardiovascular disease and osteoarthritis of knees, hips, and hands	86	1.448	0.850	2.464	0.173

A	Chronic respiratory disease	297	1.120	0.879	1.428	0.358
B	Rheumatoid arthritis	126	1.114	0.775	1.600	0.560
A and B	Chronic respiratory disease and rheumatoid arthritis	24	1.778	0.710	4.454	0.219

A	Diabetes mellitus	217	1.638	1.243	2.159	0.000
B	Rheumatoid arthritis	137	1.156	0.817	1.637	0.413
A and B	Diabetes mellitus and rheumatoid arthritis	13	1.928	0.549	6.773	0.306

A	Diabetes mellitus	218	1.647	1.251	2.169	0.000
B	Persistent injury due to an accident	120	1.078	0.743	1.565	0.692
A and B	Diabetes mellitus and persistent injury due to an accident	12	1.821	0.504	6.580	0.360

A	Cardiovascular disease	259	1.327	1.027	1.715	0.030
B	Inflammatory joint disease	262	1.077	0.832	1.395	0.572
A and B	Cardiovascular disease and inflammatory joint disease	40	1.377	0.672	2.821	0.382

A	Chronic respiratory disease	274	1.118	0.868	1.439	0.388
B	Cardiovascular disease	252	1.336	1.030	1.731	0.029
A and B	Chronic respiratory disease and cardiovascular disease	47	1.231	0.628	2.413	0.546

A	Cardiovascular disease	262	1.379	1.069	1.779	0.013
B	Diabetes mellitus	193	1.710	1.277	2.290	0.000
A and B	Cardiovascular disease and diabetes mellitus	37	0.827	0.391	1.750	0.619

A	Diabetes mellitus	157	1.651	1.195	2.282	0.002
B	Osteoarthritis of knees, hips, and hands	707	1.050	0.885	1.247	0.575
A and B	Diabetes mellitus and osteoarthritis of knees, hips, and hands	73	1.078	0.602	1.930	0.800

^*∗*^ *p* ≤ 0.005; ^+^ *p* ≤ 0.01.

**Table 5:** All possible chronic disease pairs (*N* = 105).

Number	Chronic disease pair
1	Chronic respiratory disease and cardiovascular disease
2	Chronic respiratory disease and severe bowel disease
3^*∗*^	Chronic respiratory disease and liver disease
4	Chronic respiratory disease and severe kidney disease
5	Chronic respiratory disease and diabetes mellitus
6	Chronic respiratory disease and cancer
7^*∗*^	Chronic respiratory disease and epilepsy
8	Chronic respiratory disease and migraine
9	Chronic respiratory disease and neurological disorders and stroke
10	Chronic respiratory disease and inflammatory joint disease
11	Chronic respiratory disease and rheumatoid arthritis
12	Chronic respiratory disease and osteoarthritis of knees, hips, and hands
13	Chronic respiratory disease and severe back problems
14	Chronic respiratory disease and persistent injury due to an accident
15^*∗*^	Cardiovascular disease and severe bowel disease
16^*∗*^	Cardiovascular disease and liver disease
17^*∗*^	Cardiovascular disease and severe kidney disease
18	Cardiovascular disease and diabetes mellitus
19	Cardiovascular disease and cancer
20^*∗*^	Cardiovascular disease and epilepsy
21	Cardiovascular disease and migraine
22	Cardiovascular disease and neurological disorders and stroke
23	Cardiovascular disease and inflammatory joint disease
24	Cardiovascular disease and rheumatoid arthritis
25	Cardiovascular disease and osteoarthritis of knees, hips, and hands
26	Cardiovascular disease and severe back problems
27	Cardiovascular disease and persistent injury due to an accident
28^*∗*^	Severe bowel disease and liver disease
29^*∗*^	Severe bowel disease and severe kidney disease
30^*∗*^	Severe bowel disease and diabetes mellitus
31^*∗*^	Severe bowel disease and cancer
32^*∗*^	Severe bowel disease and epilepsy
33	Severe bowel disease and migraine
34^*∗*^	Severe bowel disease and neurological disorders and stroke
35	Severe bowel disease and inflammatory joint disease
36^*∗*^	Severe bowel disease and rheumatoid arthritis
37	Severe bowel disease and osteoarthritis of knees, hips, and hands
38	Severe bowel disease and severe back problems
39^*∗*^	Severe bowel disease and persistent injury due to an accident
40^*∗*^	Liver disease and severe kidney disease
41^*∗*^	Liver disease and diabetes mellitus
42^*∗*^	Liver disease and cancer
43^*∗*^	Liver disease and epilepsy
44^*∗*^	Liver disease and migraine
45^*∗*^	Liver disease and neurological disorders and stroke
46^*∗*^	Liver disease and inflammatory joint disease
47^*∗*^	Liver disease and rheumatoid arthritis
48^*∗*^	Liver disease and osteoarthritis of knees, hips, and hands
49^*∗*^	Liver disease and severe back problems
50^*∗*^	Liver disease and persistent injury due to an accident
51^*∗*^	Severe kidney disease and diabetes mellitus
52^*∗*^	Severe kidney disease and cancer
53^*∗*^	Severe kidney disease and epilepsy
54^*∗*^	Severe kidney disease and migraine
55^*∗*^	Severe kidney disease and neurological disorders and stroke
56^*∗*^	Severe kidney disease and inflammatory joint disease
57^*∗*^	Severe kidney disease and rheumatoid arthritis
58	Severe kidney disease and osteoarthritis of knees, hips, and hands
59^*∗*^	Severe kidney disease and severe back problems
60^*∗*^	Severe kidney disease and persistent injury due to an accident
61	Diabetes mellitus and cancer
62^*∗*^	Diabetes mellitus and epilepsy
63^*∗*^	Diabetes mellitus and migraine
64^*∗*^	Diabetes mellitus and neurological disorders and stroke
65	Diabetes mellitus and inflammatory joint disease
66	Diabetes mellitus and rheumatoid arthritis
67	Diabetes mellitus and osteoarthritis of knees, hips, and hands
68	Diabetes mellitus and severe back problems
69	Diabetes mellitus and persistent injury due to an accident
70^*∗*^	Cancer and epilepsy
71^*∗*^	Cancer and migraine
72^*∗*^	Cancer and neurological disorders and stroke
73	Cancer and inflammatory joint disease
74^*∗*^	Cancer and rheumatoid arthritis
75	Cancer and osteoarthritis of knees, hips, and hands
76	Cancer and severe back problems
77^*∗*^	Cancer and persistent injury due to an accident
78^*∗*^	Epilepsy and migraine
79^*∗*^	Epilepsy and neurological disorders and stroke
80^*∗*^	Epilepsy and inflammatory joint disease
81^*∗*^	Epilepsy and rheumatoid arthritis
82^*∗*^	Epilepsy and osteoarthritis of knees, hips, and hands
83^*∗*^	Epilepsy and severe back problems
84^*∗*^	Epilepsy and persistent injury due to an accident
85^*∗*^	Migraine and neurological disorders and stroke
86	Migraine and inflammatory joint disease
87	Migraine and rheumatoid arthritis
88	Migraine and osteoarthritis of knees, hips, and hands
89	Migraine and severe back problems
90	Migraine and persistent injury due to an accident
91	Neurological disorders and stroke and inflammatory joint disease
92^*∗*^	Neurological disorders and stroke and rheumatoid arthritis
93	Neurological disorders and stroke and osteoarthritis of knees, hips, and hands
94	Neurological disorders and stroke and severe back problems
95^*∗*^	Neurological disorders and stroke and persistent injury due to an accident
96	Inflammatory joint disease and rheumatoid arthritis
97	Inflammatory joint disease and osteoarthritis of knees, hips, and hands
98	Inflammatory joint disease and severe back problems
99	Inflammatory joint disease and persistent injury due to an accident
100	Rheumatoid arthritis and osteoarthritis of knees, hips, and hands
101	Rheumatoid arthritis and severe back problems
102^*∗*^	Rheumatoid arthritis and persistent injury due to an accident
103	Osteoarthritis of knees, hips, and hands and severe back problems
104	Osteoarthritis of knees, hips, and hands and persistent injury due to an accident
105	Severe back problems and persistent injury due to an accident

^*∗*^DUOs excluded because *N* ≤ 10.
